# Organocatalysis in heterocyclic synthesis: DABCO as a mild and efficient catalytic system for the synthesis of a novel class of quinazoline, thiazolo [3,2-*a*]quinazoline and thiazolo[2,3-*b*] quinazoline derivatives

**DOI:** 10.1186/1752-153X-7-82

**Published:** 2013-05-07

**Authors:** Haider Behbehani, Hamada Mohamed Ibrahim

**Affiliations:** 1 Chemistry Department, Faculty of Science, Kuwait University, P.O. Box 5969, Safat 13060, Kuwait; 2 Chemistry Department, Faculty of Science, Fayoum University, Fayoum 63514, A. R, Egypt

**Keywords:** Organocatalysis, DABCO, Quinazoline, Thiazolo[3,2-*a*] quinazoline, Thiazolo[2,3-*b*]quinazoline

## Abstract

**Background:**

There are only limited publications devoted to the synthesis of especially thiazolo[3,2-*a*]quinazoline which involved reaction of 2-mercaptopropargyl quinazolin-4-one with various aryl iodides catalyzed by Pd-Cu or by condensation of 2-mercapto-4-oxoquinazoline with chloroacetic acid, inspite of this procedure was also reported in the literature to afford the thiazolo [2,3-*b*] quinazoline. So the multistep synthesis of the thiazolo[3,2-*a*]- quinazoline suffered from some flaws and in this study we have synthesized a novel class of thiazoloquinazolines by a simple and convenient method involving catalysis by 1,4-diazabicyclo[2.2.2]octane (DABCO).

**Results:**

A new and convenient one-pot synthesis of a novel class of 2-arylidene-2*H*-thiazolo[3,2-*a*]quinazoline-1,5-diones **9a-i** was established through the reaction between methyl-2-(2-thio-cyanatoacetamido)benzoate (**4**) and a variety of arylidene malononitriles **8a-i** in the presence of DABCO as a mild and efficient catalytic system *via* a Michael type addition reaction and a mechanism for formation of the products observed is proposed. Moreover **4** was converted to ethyl-2-[(4-oxo-3,4-dihydroquinazolin-2-yl)thio]acetate (**10**) upon reflux in ethanol containing DABCO as catalyst. The latter was reacted with aromatic aldehydes and dimethylformamide dimethylacetal (DMF-DMA) to afford a mixture of two regioselectively products with identical percentage yield, these two products were identified as thiazolo[3,2-*a*]quinazoline **9,13** and thiazolo[2,3-*b*]quinazoline **11,12** derivatives respectively. The structure of the compounds prepared in this study was elucidated by different spectroscopic tools of analyses also the X-ray single crystal technique was employed in this study for structure elucidation, Z/E potential isomerism configuration determination and to determine the regioselectivity of the reactions.

**Conclusion:**

A simple and efficient one-pot synthesis of a novel class of 2-arylidene-2*H*-thiazolo[3,2-*a*]quinazoline-1,5-diones **9a-i** was established through DABCO catalyzed Michael type addition reaction. In addition many fused quinazoline and quinazoline derivatives were synthesized which appeared as valuable precursors in synthetic and medicinal chemistry.

## Background

The synthesis of fused heterocycles has attracted considerable interest in heterocyclic chemistry as the fusion of biodynamic heterosystems has proved to be a very attractive and useful for the design of new molecular framework of potential drugs with varying pharmacological activities. A major challenge of the modern synthetic chemistry is to design highly efficient chemical reaction sequences which provide molecules containing maximum complexity and structural diversity with interesting bioactivities in minimum number of synthetic steps. Recently, organocatalysis has increased spectacularly in the last few years as a result of both the novelty of the concept and, more importantly, the fact that the efficiency and the selectivity of many organocatalytic reactions meet the standards of established organic reactions. One of these organocatalysts is the 1,4-diazabicyclo[2.2.2]octane (DABCO) which has received considerable attention as an inexpensive, eco-friendly, high reactive and non-toxic base catalyst for various organic transformations, affording the corresponding products in excellent yields with high selectivity [1-5]. We have found that the quinazolines and condensed quinazolines are versatile classes of fused heterocycles that are of considerable interest because of the diverse range of their biological properties and the potent pharmacological activities such as anticancer [6,7], antitumor [8,9], anti-oxidant [10], analgesic [11], anti-inflammatory [7,12], anti-convulsant [13], anti HIV, antibacterial, antifungal [14-17], antihypertensive [18], antileishmanial [19] and CNS depressant activity [20]. On the other hand, the considerable biological and medicinal activities of the thiazoles and their derivatives have also attracted continuing interest over the years because of their varied biological activities exemplified as antibacterial, antifungal [21-24], antitubercular [25], anticancer [26-28], antidiabetic [29], anti HIV [30] in addition to large applications in the drug development for the treatment of many disease. So, on the basis of the above findings the quinazoline and thiazole are privileged structures, which attracted considerable attention in the designing of biologically active molecules and combining them in one molecule exemplified by the thiazoloquinazoline system it is expected to furnish biologically active molecule with characteristic features. In the last decade numerous methods have been developed for the synthesis of highly substituted thiazoloquinazoline system exemplified by thiazolo[2,3-*b*]quinazoline [8,31-33], thiazolo[5,4-*f*]quinazoline [34,35], thiazolo[4,5-*h*]quinazolin [36], thiazolo[5,4-*c*]quinoline [37], thiazolo[4,3-*b*]quinazoline [38] and thiazolo[3,2-*a*]quinazoline [39]. However, after detailed literature survey it was observed that there were only limited publications devoted to the synthesis of especially thiazolo[3,2-*a*]quinazoline which involved reaction of 2-mercaptopropargyl quinazolin-4-one with various aryl iodides catalyzed by Pd-Cu [39] or by condensation of 2-mercapto-4-oxoquinazoline with chloroacetic acid [40], inspite of this procedure was also reported in the literature to afford the thiazolo[2,3-*b*]quinazoline. So the multistep synthesis of thiazoloquinazolines especially thiazolo[3,2-*a*]quinazoline suffered from some flaws and in continuation of our research program on the synthesis of nitrogen and sulphur containing novel heterocycles [41-43] of pharmaceutical interest and in view of the operational simplicity in this study we have synthesized a novel class of thiazoloquinazolines by a simple and convenient method involving catalysis by DABCO. The X-ray single crystal technique as an advanced tool of analysis was employed in this study for structure elucidation and for determination the regioselectivety of the reactions.

## Results and discussion

### Synthetic chemistry

**Scheme 1:**
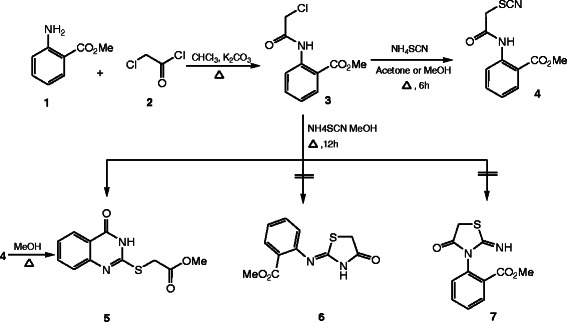
Synthesis of methyl-2-(2-thiocyanatoacetamido)benzoate (4) and the quinazoline derivative 5.

**Scheme 2:**
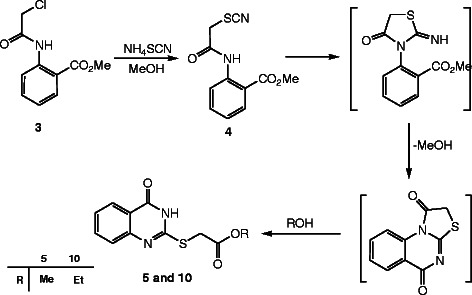
The mechanistic pathway for compounds 5 and 10.

**Figure 1:**
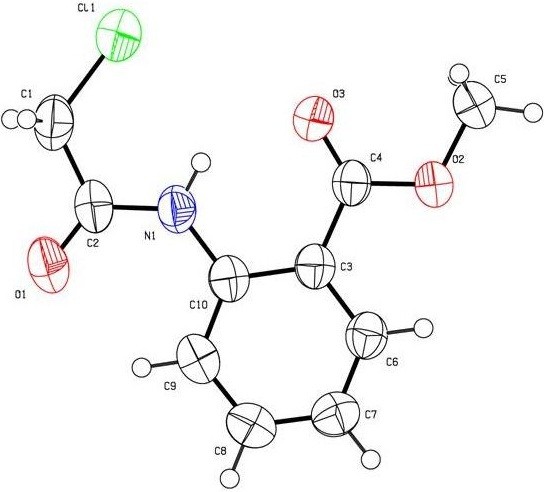
**X-ray single crystal structure determined for compound 3 (CCDC 916675)**[46]**.**

**Figure 2:**
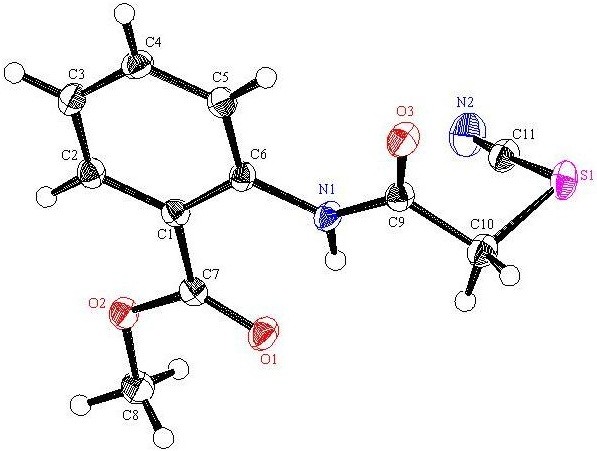
**X-ray single crystal structure determined for compound 4 (CCDC 916665)**[47]**.**

**Figure 3:**
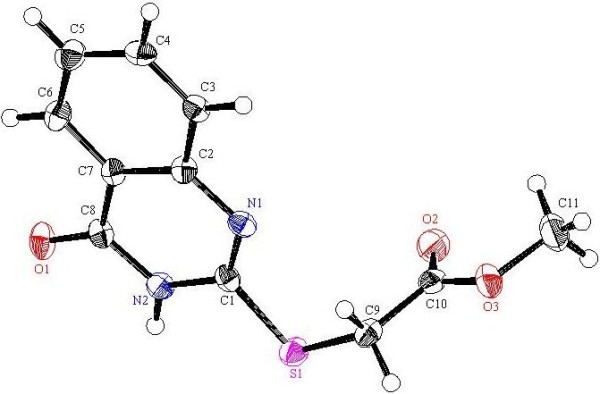
**X-ray single crystal structure determined for compound 5 (CCDC 916666)**[48]**.**

The synthetic strategy of our study to obtain the targeted compounds began by preparing the starting material methyl-2-(2-thiocyanatoacetamido)benzoate (**4**) which prepared in two synthetic steps firstly by reacting the methyl anthranilate (MA) (**1**) with chloroacetyl chloride (**2**) to afford methyl-2-(2-chloroacetamido)benzoate (**3**) and secondly by reacting the latter with ammonium thiocyanate in refluxing acetone. Moreover conducting the reaction between the methyl-2-(2-chloroaceta-mido) benzoate (**3**) and the ammonium thiocyanate in absolute methanol afford two products depending on the refluxing time, **4** was formed after 6h while the methyl-2-[(4-oxo-3,4-dihydroquinazolin-2-yl)thio]acetate (**5**) was formed after 12 h and not compound **6** or **7**[43,44]. Also compound **5** can be obtained by refluxing the formed methyl-2-(2-thiocyanatoacetamido)benzoate (**4**) in methanol [45] (cf. Schemes 1 and 2). The structure of compounds **3**, **4** and **5** was confirmed through the X-ray single crystal structure determination (cf. Figures 1, 2, 3).

**Figure 4:**
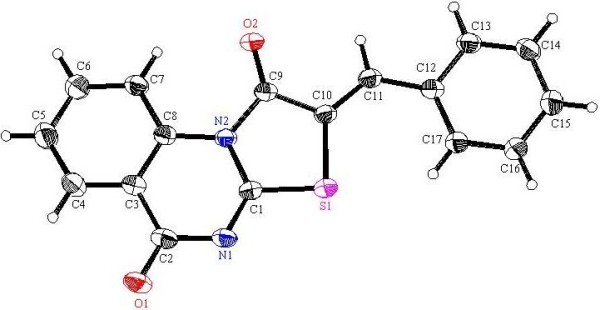
**X-ray single crystal structure determined for compound 9a (CCDC 916667)**[49]**.**

**Figure 5:**
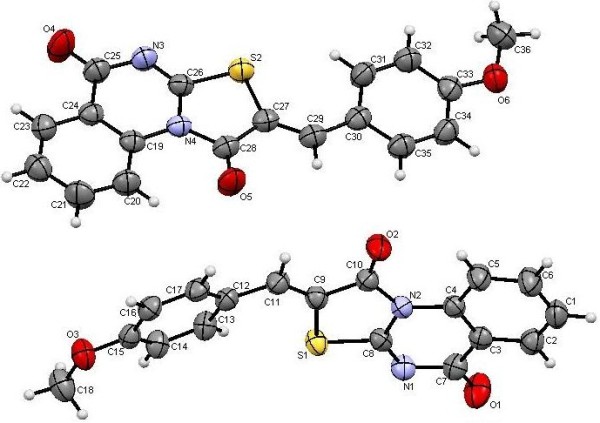
**X-ray single crystal structure determined for compound 9c (CCDC 916674)**[50]**.**

**Figure 6:**
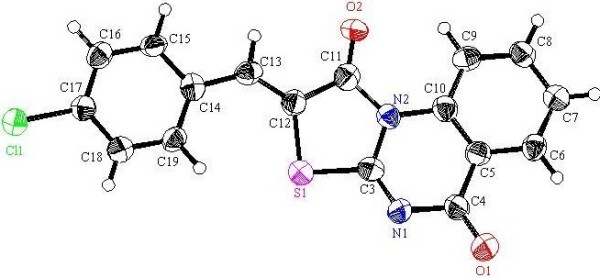
**X-ray single crystal structure determined for compound 9d (CCDC 916671)**[51]**.**

**Figure 7:**
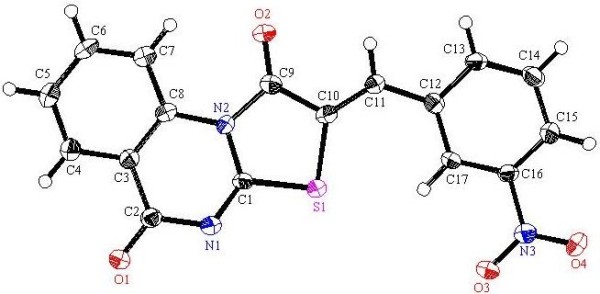
**X-ray single crystal structure determined for compound 9f (CCDC 916670)**[52]**.**

**Figure 8:**
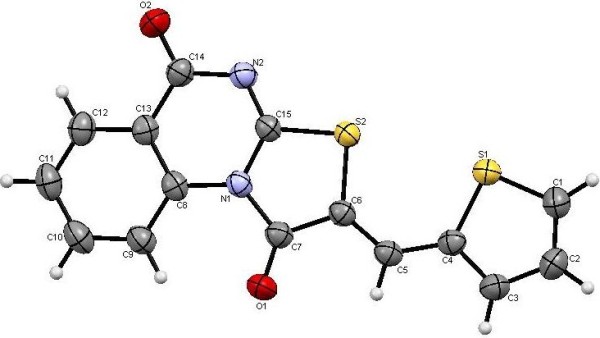
**X-ray single crystal structure determined for compound 9h (CCDC 916673)**[53]**.**

**Scheme 3:**
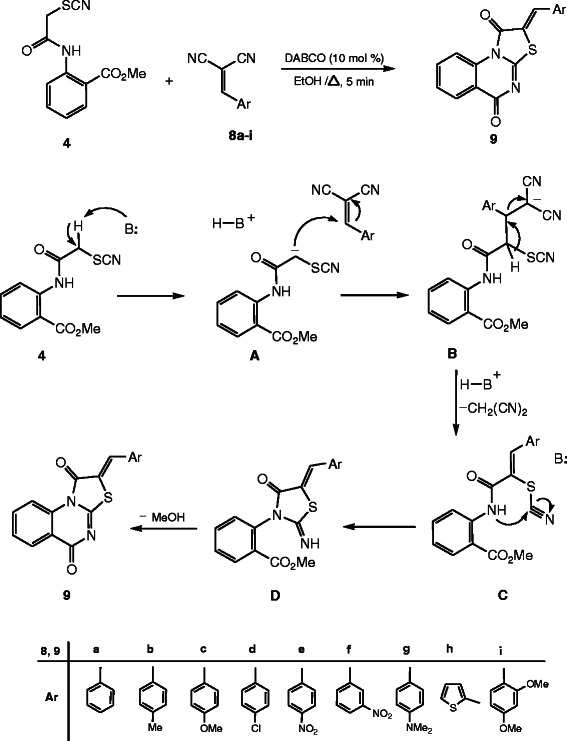
**Synthesis and the mechanistic pathway for the thiazolo[3,2-*a*]quinazoline derivatives 9.**

Now it was of interest to explore the scope and limitations and generality of the methyl-2-(2-thiocyanatoacetamido)benzoate (**4**) as a precursor for the synthesis of some polyfunctionally substituted fused thiazoloquinazoline derivatives for which we might expect a wide spectrum of bioresponses. Thus the active methylene in the methyl-2-(2-thiocyanatoacetamido)benzoate (**4**) underwent nucleophilic addition reaction to the double bond of a variety of arylidene malononitriles **8a-i***via* a Michael type addition reaction [42], by refluxing in ethanol containing 10 mol % of DABCO as catalyst to give a substance whose structure was determined as thiazolo[3,2-*a*]quinazoline derivatives **9a-i**, as established from the accurate mass determination, ^1^H-NMR and ^13^C-NMR. Moreover this structure was also confirmed through the X-ray single crystal structure determination for **9a**, **9c**, **9d**, **9f** and **9h** (cf. Figures 4, 5, 6, 7, 8 and Scheme 3). It is worth mention that the short reaction times, easy workup, very good to excellent yields, and mild reaction conditions make this Michael type addition reaction followed by interamolecular cyclization both practical and attractive. It is believed that initially the carbanion which formed from **4** by the action of the base (B:) DABCO undergoes nucleophilic addition to the double bond of the arylidene malononitrile to form the adduct **B** followed by losing one molecule of malononitrile and subsequent interamolecular cyclization forming the thiazolidinone ring *via* attack of the NH moiety at SCN, and finally the thiazolo[3,2-*a*]quinazoline derivatives **9** was formed through losing one molecule of methanol during the another interamolecular cyclization. Also in order to confirm the above synthetic route we try to separate one of the formed intermediates during the reaction and we success to isolate the intermediate **D** [Ar = *P*-NO_2_C_6_H_4_] during the preparation of **9e**. The X-ray single crystal technique was successfully employed in this study to confirm the potential formation of the Z isomer during the preparation of **9** as a sole isolable isomer product.

To optimize the reaction conditions, we systematically investigated the reaction parameters using **4** and benzylidene malononitriles **8a** (Table 1). First, the effect of bases was investigated (entries 1–6). It was found that the DABCO afford **9a** in very good yields, whereas other bases, such as piperidine, morpholine, DBU, L-proline and K_2_CO_3_ were less effective. Then we probed the influence of different solvents on the reaction (entries 7–10). EtOH was found to be an effective solvent for good results. CH_3_CN, DMF, dioxane and MeOH were found to be less effective. With the optimized reaction conditions in hand, we then explored the scope and generality of the synthesizing thiazolo[3,2-*a*]quinazoline derivatives **9***via* Michael type addition reaction followed by the interamolecular cyclization. In addition we optimized the quantity of the catalyst added, as we found that 10 mol % of DABCO is the best quantity for the reaction yield and we found that as the quantity of the added catalyst increased or decreased the yield of the reaction become lowered (Table 2).

**Table 1:** **Optimization of conditions of the synthesis 9a **^**a**^

**Entry **^a^	**Base**	**Solvent**	**Time(min)**	**Yield (%)**
1	DABCO	EtOH	5	81
2	piperidine	EtOH	30	67
3	morpholine	EtOH	25	61
4	DBU	EtOH	15	55
5	L-proline	EtOH	40	69
6	K_2_CO_3_	EtOH	60	none
7	DABCO	CH_3_CN	15	35
8	DABCO	DMF	5	46
9	DABCO	Dioxane	5	51
10	DABCO	MeOH	5	68

^a^ Reaction conditions: methyl-2-(2-thiocyanatoacetamido)benzoate (**4**) (5 mmol), benzylidene malononitriles **8a** (5 mmol), and base (10 mol %) in solvent (25 mL) under reflux for the given time.

**Table 2:** Optimization mol % of DABCO during the synthesis 9a

**Entry **^**a**^	**mol % of DABCO**	**Yield (%)**
1	5	75
2	10	81
3	15	73
4	20	70
5	30	62
6	40	54
7	50	46
8	60	45
9	70	45

**Scheme 4:**
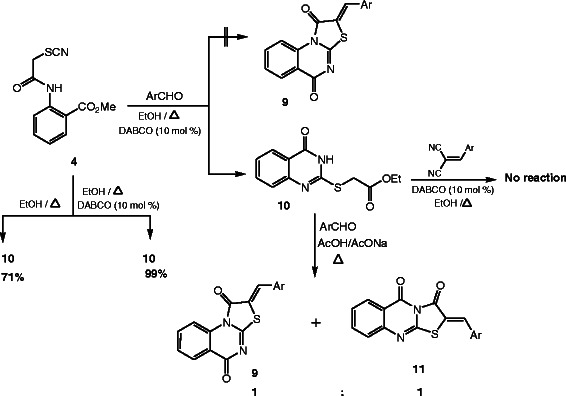
Reaction of 10 with aromatic aldehydes.

**Figure 9:**
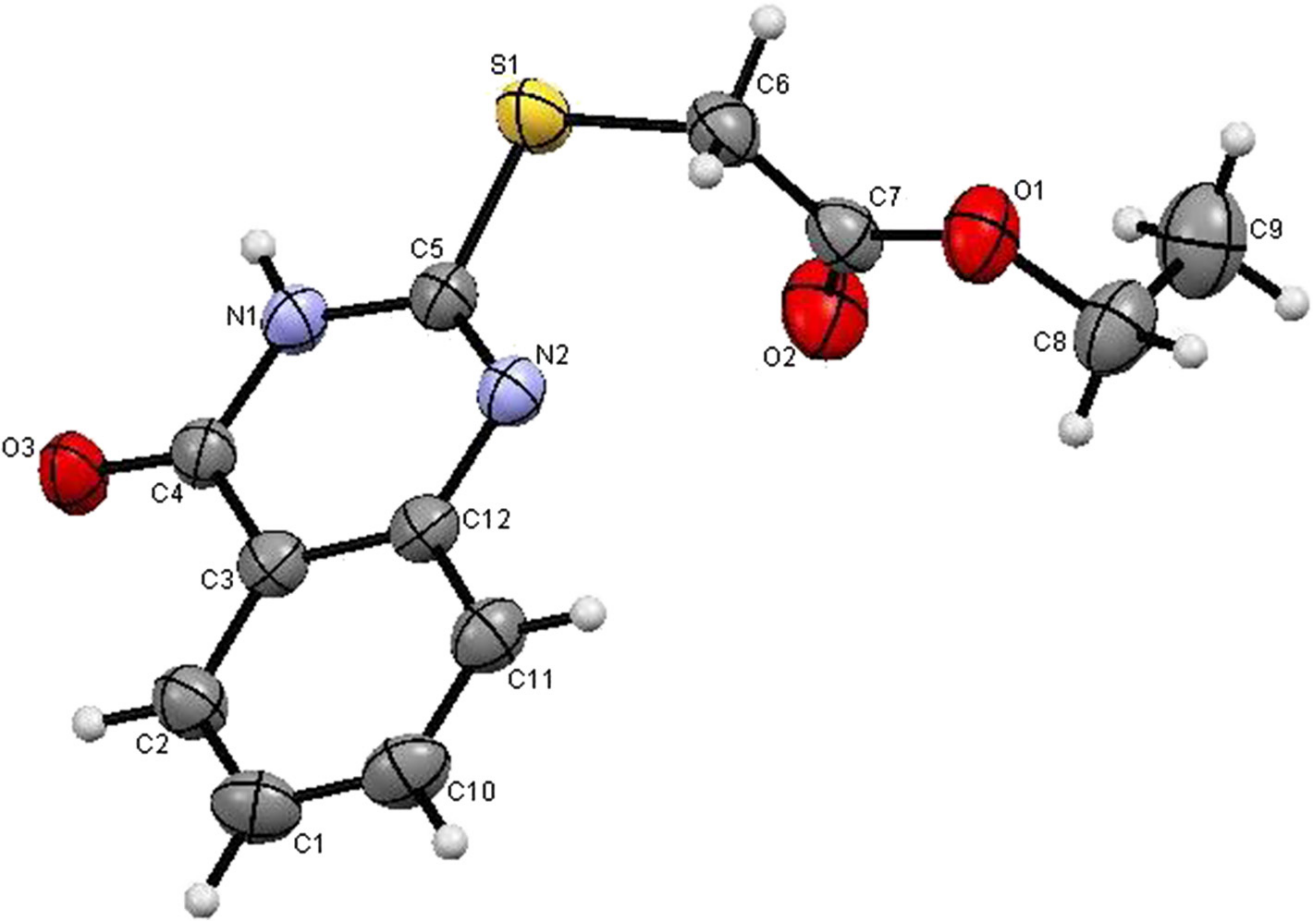
**X-ray single crystal structure determined for compound 10 (CCDC 916672)**[54]**.**

In order to generate an alternative route for the synthesis of the above thiazolo[3,2-*a*]quinazoline **9** we conduct a reaction between methyl-2-(2-thiocyanatoacetamido)benzoate (**4**) and aromatic aldehydes under the same reaction conditions (EtOH, DABCO), but the structure of the obtained product was identified as ethyl-2-[(4-oxo-3,4-dihydroquinazolin-2-yl)thio]acetate (**10**) [45] and not as **9**, based on the spectrometric analyses and from the X-ray single crystal structure determination (cf. Scheme 4, Figure 9). Moreover refluxing **4** in ethanol only give **10** in 71% yields while refluxing it in ethanol containing DABCO affords **10** in 99% yields so the presence of the DABCO enhance the reaction yield. Also the solvent has an effect on the reaction product since refluxing **4** in ethanol give **10** while refluxing **4** in methanol give **5** (cf. Scheme 2). Although the reaction between **10** and arylidene malononitriles does not take place conducting reaction between **10** and aromatic aldehydes in acetic acid containing sodium acetate afforded two regioselectively products with identical percentage yield as indicated from the ^1^H-NMR spectra, these two products were identified as **9** and **11** respectively, this mean that the regioselectively for this reaction is 50% for each route of cyclization.

**Scheme 5:**
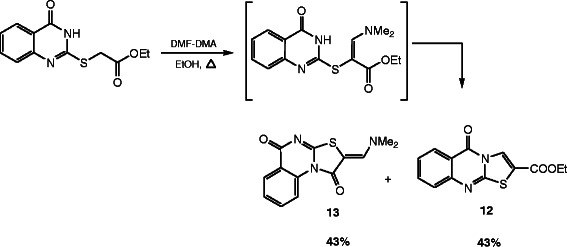
Reaction of 10 with dimethylformamide dimethylacetal (DMF-DMA).

**Figure 10:**
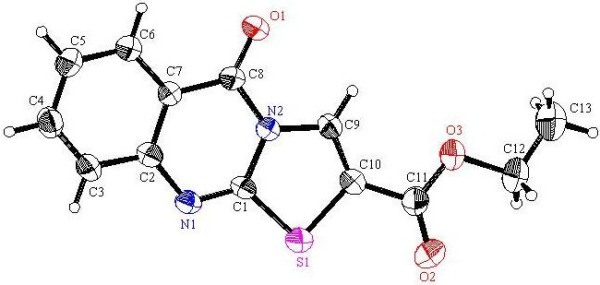
**X-ray single crystal structure determined for compound 12 (CCDC 916668)**[55]**.**

**Figure 11:**
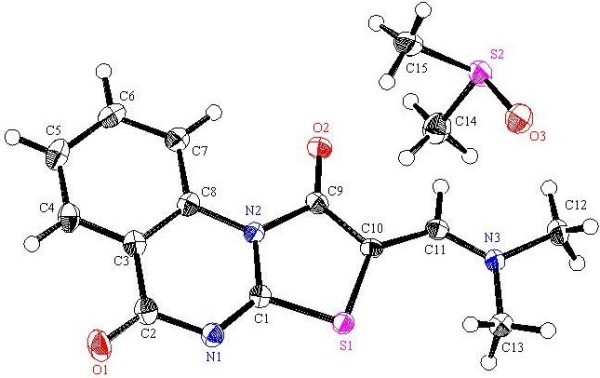
**X-ray single crystal structure determined for compound 13 (CCDC 916669)**[56]**.**

The obtained ethyl-2-[(4-oxo-3,4-dihydroquinazolin-2-yl)thio]acetate (**10**) seem interesting precursor for the synthesis of a variety of a novel quinazoline and fused quinazoline derivatives. Thus reacting **10** with dimethylformamide dimethylacetal (DMF-DMA) yield also two regioselectively products with identical percentage yield, these two products were identified as **12** and **13** respectively, the structure of these two products was confirmed *via* the X-ray single crystal determination (cf. Scheme 5, Figures 10, 11).

**Scheme 6:**
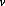
Synthesis of some quinazoline derivatives.

Moreover reacting **10** with hydrazine hydrate in refluxing ethanol affording the corresponding hydrazide derivatives **14** which on reaction with aromatic aldehydes affording the corresponding condensation product **15**, also **10** reacts with primary aromatic amines to afford the corresponding acetamide derivatives **16**. In addition the triazolylquinazoline derivative **18** was formed *via* reaction of the hydrazide derivatives **14** with carbon disulfide in alcoholic potassium hydroxide to form **17** which on reaction with hydrazine hydrate affords the triazole **18**. Moreover the quinazoline ethyl ester **10** was reacted with ethanol amine to afford the corresponding quinazoline hydroxyl derivative **19** (cf. Scheme 6).

#### Experimental

### General

Melting points were recorded on a Griffin melting point apparatus and are reported uncorrected. IR spectra were recorded using KBr disks using a Perkin-Elmer System 2000 FT-IR spectrophotometer. ^1^H-NMR (400 MHz) or (600 MHz) and ^13^C-NMR (100 MHz) or (150 MHz) spectra were recorded at 25°C in CDCl_3_ or DMSO-*d*_*6*_ as solvent with TMS as internal standard on a Bruker DPX 400 or 600 super-conducting NMR spectrometer. Chemical shifts are reported in ppm. Mass spectra and HRMS were measured using a high resolution GC-MS (DFS) thermo spectrometers with EI (70 EV). Follow up of the reactions and checking homogeneity of the prepared compounds was made by thin layer chromatography (TLC). The crystal structures were determined by a Rigaku R-AXIS RAPID diffractometer and Bruker X8 Prospector and the data were collected at a temperature of 20 ± 1°C to a maximum 2θ value of 55.0° or 66.61° using the ω scanning technique. The structure was solved either by direct method using SHELXS-97 (Sheldrick, 2008) and refined by Full-matrix least-squares on F^2^. The non-hydrogen atoms were refined anisotropically. Data were corrected for absorption effects using the multi-scan method (SADABS) or by charge flipping method and expanded using Fourier techniques. The non-hydrogen atoms were refined anisotropically. Hydrogen atoms were refined using the riding model

#### Methyl-4-(2-chloroacetamido)benzoate (3)

A solution of methyl anthranilate (**1**) (10 mmol, 1.52 g) and chloroacetyl chloride (**2**) (1.36 g, 12 mmol) in chloroform (50 mL) was refluxed in the presence of K_2_CO_3_ (2.1 g, 15 mmole) for 3 h. Then the solvent was removed in *vacuo* and the residue was stirred with water (100 mL) and filtered. The solid product is then washed with 5% NaHCO_3_ solution and subsequently with water. The crude product was dried and recrystallized from EtOH as white crystals, yield: 96%, m.p. 90-91°C; IR (KBr): v/cm^−1^ 3225 (NH), 1699, 1679 (2CO); ^1^H-NMR (DMSO-*d*_*6*_): *δ* = 3.89 (s, 3H, CH_3_), 4.45 (s, 2H, CH_2_), 7.25 (t, *J* = 8.0 Hz, 1H , Ar-H), 7.66 (t, *J* = 8.0 Hz, 1H, Ar-H), 7.98 (d, *J* = 8.0 Hz, 1H, Ar-H), 8.40 (d, *J* = 8.0 Hz, 1H, Ar-H) and 11.34 ppm (s, 1H, NH); ^13^C-NMR (DMSO-*d*_*6*_): *δ* = 43.3 (CH_2_), 52.5 (CH_3_), 116.8, 120.4, 123.7, 130.7, 134.2, 139.2, 165.1 and 167.4 ppm (Ar-C and CO); MS (EI): *m/z* (%) 229 (M^+^+2, 14.12), 228 (M^+^+1, 7.85), 227 (M^+^, 43.55); HRMS (EI): *m/z* calcd. for C_10_H_10_^35^ClNO_3_ (M^+^) 227.0343, found 227.0343. Crystal data: C_10_H_10_ClNO_3_, M = 227.65, monoclinic, a = 12.8980(12) Å, b = 4.6089(4) Å, c = 18.2514(15) Å, V = 1031.08(16) Å^3^, α = γ = 90.00°, β = 108.133(5), space group: P 1 21/c 1, Z = 4, D_calc_ = 1.466 Mg cm^−3^ , No. of reflections measured 4589, 2θ_max_ = 66.59°, R1 = 0.034. Figure 1 illustrates the structure as determined. Full data can be obtained on request from the CCDC [46].

#### Methyl-2-(2-thiocyanatoacetamido)benzoate (4)

A solution of methyl-4-(2-chloroacetamido)benzoate (**3**) (2.27 g, 10 mmol) and ammonium thiocyanate (15 mmol) in acetone or absolute methanol (30 mL) was refluxed for 6 h and allowed to cool. The formed precipitate was filtered off, washed with water and then recrystallised from MeOH as white crystals, yield: 92%, m.p. 109–110°C; IR (KBr): v/cm^−1^ 3241 (NH), 2155 (SCN), 1737, 1698 (2CO); ^1^H-NMR (DMSO-*d*_*6*_): *δ* = 3.87 (s, 3H, CH_3_), 4.24 (s, 2H, CH_2_), 7.25 (t, *J* = 8.0 Hz, 1H, Ar-H), 7.63 (t, *J* = 8.0 Hz, 1H, Ar-H), 7.91 (d, *J* = 8.0 Hz, 1H, Ar-H), 8.16 (d, *J* = 8.0 Hz, 1H, Ar-H) and 10.93 ppm (s, 1H, NH); ^13^C-NMR (DMSO-*d*_*6*_): *δ* = 37.4 (CH_2_), 52.5 (CH_3_), 112.7 (S*CN*), 118.8, 121.7, 124.1, 130.6, 133.9, 138.4, 164.6 and 167.2 ppm (Ar-C and CO); MS (EI): *m/z* (%) 251 (M^+^+1, 10.42), 250 (M^+^, 34.91); HRMS (EI): *m/z* calcd. for C_11_H_10_N_2_O_3_S (M^+^) 250.0406, found 250.0407. Crystal data: C_11_H_10_N_2_O_3_S, M = 250.28, triclinic, a = 6.7349(6) Å, b = 8.3058(9) Å, c = 10.3398(8) Å, V = 558.19(9) Å^3^, α =86.526(6), β = 85.256(6)°, γ = 75.747(6), space group: P-1 (#2), Z = 2, D_calc_ = 1.489 Mg cm^−3^, No. of reflections measured 2518, 2θ_max_ =54.9°, R1 = 0.033. Figure 2 illustrates the structure as determined. Full data can be obtained on request from the CCDC [47].

#### Methyl-2-(4-oxo-3,4-dihydroquinazolin-2-ylthio)acetate (5)

A solution of methyl-4-(2-chloroacetamido)benzoate (**3**) (2.27 g, 10 mmol) and ammonium thiocyanate (15 mmol) in methanol (30 mL) was refluxed for 12 h, or refluxing 4 in MeOH for 6h then the reaction mixture was allowed to cool down to room temperature. The formed precipitate was filtered off, washed with water and then recrystallised from MeOH as white crystals, yield: 88%, m.p. 191–192°C; IR (KBr): v/cm^−1^ 3172 (NH), 1737, 1686 (2CO); ^1^H-NMR (DMSO-*d*_*6*_): *δ* = 3.69 (s, 3H, CH_3_), 4.11 (s, 2H, CH_2_), 7.41-7.45 (m, 2H , Ar-H), 7.76 (t, *J* = 7.6 Hz, 1H, Ar-H), 8.03 (d, *J* = 7.6 Hz, 1H, Ar-H), 12.72 ppm (s, 1H, NH); ^13^C-NMR (DMSO-*d*_*6*_): *δ* = 32.1 (CH_2_), 52.4 (CH_3_), 119.8, 125.8, 125.9, 126.0, 134.7, 148.1, 154.6, 161.1 and 169.0 ppm (Ar-C and CO); MS (EI): *m/z* (%) 251 (M^+^+1, 7.24), 250 (M^+^, 32.55); HRMS (EI): *m/z* calcd. for C_11_H_10_N_2_O_3_S (M^+^) 250.0406, found 250.0406. Crystal data: C_11_H_10_N_2_O_3_S, M = 250.28, orthorhombic, a = 17.863(2) Å, b = 13.1670(7) Å, c = 4.6272(2) Å, V = 1088.3(1) Å^3^, α = β = γ = 90.0°, space group: Pna21 (#33), Z = 4, D_calc_ = 1.527 Mg cm^−3,^ No. of reflections measured 2058, 2θ_max_ = 54.80°, R1 = 0.0384. Figure 3 illustrates the structure as determined. Full data can be obtained on request from the CCDC [48].

#### General procedure for the synthesis of thiazolo[3,2-*a*]- quinazoline derivatives 9a–i

Independent mixtures of methyl-2-(2-thiocyanatoacetamido)-benzoate (**4**) (1.25 g, 5 mmol) and the appropriate arylidene malononitrile **8a-i** (5 mmol) in ethanol (25 mL) containing DABCO (0.11 g, 10 mol %) were stirred at reflux for 5 min. Then, the reaction mixtures were allowed to cool down to room temperature. The solid which formed was collected by filtration, washed with hot ethanol, and recrystallized from the appropriate solvent to afford **9a–i** respectively, as pure substances.

#### (*Z*)-2-Benzylidene-2*H*-thiazolo[3,2-*a*]quinazoline-1,5-dione (9a)

Recrystallized from an EtOH/ dioxane (1:1) mixture as canary yellow crystals, yield: (81%), m.p. 236–237°C; IR (KBr): v/cm^−1^ 1717, 1672 (2CO); ^1^H-NMR (DMSO-*d*_*6*_): *δ* = 7.54-7.68 (m, 4H, Ar-H), 7.76 (d, *J* = 7.6 Hz, 2H, Ar-H), 7.94 (t, *J* = 8.0 Hz, 1H, Ar-H), 8.15 (d, *J* = 8.0 Hz, 1H, Ar-H), 8.17 (s, 1H, olefinic *CH*) and 8.98 ppm (d, *J* = 8.0 Hz, 1H, Ar-H); ^13^C-NMR (DMSO-*d*_*6*_): *δ* = 116.3, 117.7, 118.8, 127.9, 127.9, 129.6, 130.5, 131.3, 132.7, 134.5, 135.8, 137.1, 164.2, 164.3 and 165.2 ppm (Ar-C and CO); MS (EI): *m/z* (%) 307 (M^+^+1, 19.44), 306 (M^+^, 100); HRMS (EI): *m/z* calcd. for C_17_H_10_N_2_O_2_S (M^+^) 306.0457, found 306.0457. Crystal data: C_17_H_10_N_2_O_2_S, M = 306.34, triclinic, a = 6.391(5) Å, b = 8.650(7) Å, c = 12.77(1)Å, V = 676.2(9) Å^3^, α =76.57(2), β =87.89(1), γ = 79.99(1)°, space group: P-1 (#2), Z = 2, D_calc_ = 1.504 Mg cm^−3^, No. of reflections measured 5937, 2θ_max_ = 52.70°, R1 = 0.0458. Figure 4 illustrates the structure as determined. Full data can be obtained on request from the CCDC [49].

#### (*Z*)-2-(4-Methylbenzylidene)-2*H*-thiazolo[3,2-*a*]quinazoline-1,5-dione (9b)

Recrystallized from dioxane as pale yellow crystals, yield: (79%), m.p. 253–254°C; IR (KBr): v/cm^−1^ 1726, 1681 (2CO); ^1^H-NMR (DMSO-*d*_*6*_): *δ* = 2.40 (s, 3H, CH_3_), 7.40 (d, *J* = 7.6 Hz, 2H, Ar-H), 7.59-7.64 (m, 3H, Ar-H), 7.90 (t, *J* = 8.0 Hz, 1H, Ar-H), 8.10 (s, 1H, olefinic *CH*), 8.16 (d, *J* = 8.0 Hz, 1H, Ar-H) and 8.97 ppm (d, *J* = 8.0 Hz, 1H, Ar-H); ^13^C-NMR (DMSO-*d*_*6*_): *δ* = 21.2 (CH_3_), 116.3, 117.5, 117.8, 127.8, 127.9, 130.1, 130.2, 130.6, 134.5, 135.9, 137.1, 141.8, 164.2, 164.4 and 165.3 ppm (Ar-C and CO); MS (EI): *m/z* (%) 321 (M^+^+1, 17.99), 320 (M^+^, 100); HRMS (EI): *m/z* calcd. for C_18_H_12_N_2_O_2_S (M^+^) 320.0613, found 320.0614.

#### (*Z*)-2-(4-Methoxybenzylidene)-2*H*-thiazolo[3,2-*a*]quinazoline-1,5-dione (9c)

Recrystallized from dioxane as yellow crystals, yield: (83%), m.p. 261–262 °C; IR (KBr): v/cm^−1^ 1721, 1676 (2CO); ^1^H-NMR (DMSO-*d*_*6*_): *δ* = 3.87 (s, 2H, O*CH*_*3*_), 7.18 (d, *J* = 7.6 Hz, 2H, Ar-H), 7.66 (t, *J* = 8.0 Hz, 1H, Ar-H), 7.75 (d, *J* = 7.6 Hz, 2H, Ar-H), 7.93 (t, *J* = 8.0 Hz, 1H, Ar-H), 8.11 (s, 1H, olefinic *CH*), 8.17 (d, *J* = 8.0 Hz, 1H, Ar-H) and 9.02 ppm (d, *J* = 8.0 Hz, 1H, Ar-H); ^13^C-NMR (DMSO-*d*_*6*_ at 80°C ): *δ* = 56.2 (O*CH*_*3*_), 115.8, 116.8, 118.6, 126.0, 128.2, 128.4, 130.9, 133.1, 134.6, 136.8, 137.6, 162.5, 164.2, 164.6 and 165.4 ppm (Ar-C and CO); MS (EI): *m/z* (%) 337 (M^+^+1, 16.96), 336 (M^+^, 100); HRMS (EI): *m/z* calcd. for C_18_H_12_N_2_O_3_S (M^+^) 336.0563, found 336.0563. Crystal data: C_18_H_12_N_2_O_3_S, M = 336.37, monoclinic, a = 8.1837(3) Å, b = 15.2696(4) Å, c = 24.0028(7) Å, V = 2996.81(16) Å^3^, α = γ = 90.00°, β = 92.397(2)°, space group: P 1 21/c 1, Z = 4, D_calc_ = 1.491 Mg cm^−3^ , No. of reflections measured 5296, 2θ_max_ = 66.73 °, R1 = 0.0339. Figure 5 illustrates the structure as determined. Full data can be obtained on request from the CCDC [50].

#### (*Z*)-2-(4-Chlorobenzylidene)-2*H*-thiazolo[3,2-*a*]quinazoline-1,5-dione (9d)

Recrystallized from dioxane as yellow crystals, yield: (85%), m.p. 279–280°C; IR (KBr): v/cm^−1^ 1720, 1692 (2CO); ^1^H-NMR (DMSO-*d*_*6*_): *δ* = 7.63-7.66 (m, 3H, Ar-H), 7.75 (d, *J* = 8.4 Hz, 2H, Ar-H), 7.92 (t, *J* = 8.4 Hz, 1H, Ar-H), 8.14 (s, 1H, olefinic *CH*), 8.20 (d, *J* = 8.4 Hz, 1H, Ar-H) and 8.97 ppm (d, *J* = 8.4 Hz, 1H, Ar-H); ^13^C-NMR (DMSO-*d*_*6*_ at 80°C ): *δ* = 116.3, 117.7, 119.6, 127.9, 129.5, 129.6, 131.6, 132.10, 134.4, 134.5, 135.8, 137.0, 164.0, 164.2 and 165.2 ppm (Ar-C and CO); MS (EI): *m/z* (%) 341 (M^+^+1, 20.28), 340 (M^+^, 100); HRMS (EI): *m/z* calcd. for C_17_H_9_^35^ClN_2_O_2_S (M^+^) 340.0067, found 340.0066. Crystal data: C_17_H_9_ClN_2_O_2_S, M = 340.78, monoclinic, a = 3.8563(7) Å, b = 12.784(2) Å, c = 28.101 (5)Å, V = 1384.9(4)Å^3^, α = γ = 90.00°, β = 91.456(7)°, space group: P21/c (#14), Z = 4, D_calc_ = 1.634 Mg cm^−3^, No. of reflections measured 7510, 2θ_max_ = 50.1°, R1 = 0.0935. Figure 6 illustrates the structure as determined. Full data can be obtained on request from the CCDC [51].

#### (*Z*)-2-(4-Nitrobenzylidene)-2*H*-thiazolo[3,2-*a*]quinazoline-1,5-dione (9e)

Recrystallized from dioxane/DMF (1:1) mixture as yellow crystals: (87%), m.p. 296–297°C; IR (KBr): v/cm^−1^ 1716, 1675 (2CO); ^1^H-NMR (DMSO-*d*_*6*_): *δ* = 7.67 (t, *J* = 8.4 Hz, 1H, Ar-H), 7.96 (t, *J* = 8.4 Hz, 1H, Ar-H), 8.01 (d, *J* = 8.4 Hz, 2H, Ar-H), 8.19 (d, *J* = 8.4 Hz, 1H, Ar-H), 8.26 (s, 1H, olefinic *CH*), 8.39 (d, *J* = 8.4 Hz, 2H, Ar-H) and 8.98 ppm (d, *J* = 8.0 Hz, 1H, Ar-H); ^13^C-NMR (DMSO-*d*_*6*_ at 80°C ): *δ* = 116.3, 117.6, 123.3, 124.5, 128.0, 128.0, 131.3, 132.8, 134.7, 136.9, 138.8, 147.8, 163.8, 163.9 and 165.1 ppm (Ar-C and CO); MS (EI): *m/z* (%) 352 (M^+^+1, 19.99), 351 (M^+^, 100); HRMS (EI): *m/z* calcd. for C_17_H_9_N_3_O_4_S (M^+^) 351.0308, found 351.0309.

#### (*Z*)-2-(3-Nitrobenzylidene)-2*H*-thiazolo[3,2-*a*]quinazoline-1,5-dione (9f)

Recrystallized from dioxane/DMF (1:2) mixture as orange crystals: (82%), m.p. 238–239°C; IR (KBr): v/cm^−1^ 1728, 1673 (2CO); ^1^H-NMR (DMSO-*d*_*6*_): *δ* = 7.66 (t, *J* = 7.8 Hz, 1H, Ar-H), 7.89 (t, *J* = 7.8 Hz, 1H, Ar-H), 7.92 (t, *J* = 8.4 Hz, 1H, Ar-H), 8.13 (d, *J* = 7.8 Hz, 1H, Ar-H), 8.19 (d, *J* = 8.4 Hz, 1H, Ar-H), 8.29 (s, 1H, olefinic *CH*), 8.33 (d, *J* = 8.4 Hz, 1H, Ar-H), 8.53 (s, 1H, Ar-H), and 8.96 ppm (d, *J* = 7.8 Hz, 1H, Ar-H); ^13^C-NMR (DMSO-*d*_*6*_ at 80°C ): *δ* = 116.3, 117.9, 122.1, 124.7, 124.9, 127.9, 128.0, 131.0, 133.4, 134.4, 134.5, 135.3, 136.9, 148.6, 163.3, 163.7 and 164.9 ppm (Ar-C and CO); MS (EI): *m/z* (%) 352 (M^+^+1, 17.78), 351 (M^+^, 100); HRMS (EI): *m/z* calcd. for C_17_H_9_N_3_O_4_S (M^+^) 351.0308, found 351.0308. Crystal data: C_17_H_9_N_3_O_4_S, M = 351.34, monoclinic, a = 8.2228(6) Å, b = 27.550(2) Å, c = 6.7703(5) Å, V = 1449.3(2) Å^3^, α = γ = 90.00°, β = 109.096(7)°, space group: P21/c (#14), Z = 4, D_calc_ = 1.610 Mg cm^−3^ , No. of reflections measured 9355, 2θ_max_ = 52.7°, R1 = 0.0482. Figure 7 illustrates the structure as determined. Full data can be obtained on request from the CCDC [52].

#### (*Z*)-2-[4-(Dimethylamino)benzylidene]-2*H*-thiazolo[3,2-*a*]-quinazoline-1,5-dione (9g)

Recrystallized from DMF as deep orange crystals: (78%), m.p. 289–290 °C; IR (KBr): v/cm^−1^ 1707, 1671 (2CO); ^1^H-NMR (DMSO-*d*_*6*_): *δ* = 3.08 (s, 6H, 2CH_3_), 6.99 (d, *J* = 9.0 Hz, 2H, Ar-H), 7.58 (d, *J* = 9.0 Hz, 2H, Ar-H), 7.63 (t, *J* = 8.4 Hz, 1H, Ar-H), 7.88 (t, *J* = 8.4 Hz, 1H, Ar-H), 8.02 (s, 1H, olefinic *CH*), 8.18 (d, *J* = 8.4 Hz, 1H, Ar-H) and 9.04 ppm (d, *J* = 8.4 Hz, 1H, Ar-H); ^13^C-NMR (DMSO-*d*_*6*_ at 80°C ): *δ* = 66.4 (2CH_3_), 110.0, 112.3, 116.3, 118.1, 119.8, 127.5, 127.8, 132.9, 133.9, 137.2, 137.5, 152.4, 163.8, 164.3 and 165.1 ppm (Ar-C and CO); MS (EI): *m/z* (%) 350 (M^+^+1, 23.97), 349 (M^+^, 100); HRMS (EI): *m/z* calcd. for C_19_H_15_N_3_O_2_S (M^+^) 349.0879, found 349.0878.

#### (*Z*)-2-(Thiophen-2-ylmethylene)-2*H*-thiazolo[3,2-*a*]-quinazoline-1,5-dione (9h)

Recrystallized from DMF as pale orange crystals: (76%), m.p. 269–270°C; IR (KBr): v/cm^−1^ 1705, 1671 (2CO); ^1^H-NMR (DMSO-*d*_*6*_): *δ* = 7.36 (t, *J* = 6.6 Hz, 1H, Ar-H), 7.65 (t, *J* = 8.4 Hz, 1H, Ar-H), 7.79 (d, *J* = 6.6 Hz, 1H, Ar-H), 7.90 (t, *J* = 8.4 Hz, 1H, Ar-H), 8.09 (d, *J* = 6.6 Hz, 1H, Ar-H), 8.18 (d, *J* = 8.4 Hz, 1H, Ar-H), 8.39 (s, 1H, olefinic *CH*) and 8.99 ppm (d, *J* = 8.4 Hz, 1H, Ar-H); ^13^C-NMR (DMSO-*d*_*6*_ at 80°C ): *δ* = 116.1, 116.3, 117.9, 127.7, 127.9, 129.0, 129.3, 134.2, 134.2, 135.5, 137.1, 137.1, 163.3, 163.8 and 165.0 ppm (Ar-C and CO); MS (EI): *m/z* (%) 313 (M^+^+1, 19.27), 312 (M^+^, 100); HRMS (EI): *m/z* calcd. for C_15_H_8_N_2_O_2_S_2_ (M^+^) 312.0021, found 312.0021. Crystal data: C_15_H_8_N_2_O_2_S_2_, M = 312.37, monoclinic, a = 3.89160(10) Å, b = 27.5592(8) Å, c = 12.1569(3) Å, V = 1293.43(6) Å^3^, α = γ = 90.00°, β = 97.2380(10)°, space group: P 1 21/n 1, Z = 4, D_calc_ = 1.604 Mg cm^−3^ , No. of reflections measured 8288, 2θ_max_ = 66.65°, R1 = 0.0320. Figure 8 illustrates the structure as determined. Full data can be obtained on request from the CCDC [53].

#### (*Z*)-2-(2,4-Dimethoxybenzylidene)-2*H*-thiazolo[3,2-*a*]-quinazoline-1,5-dione (9i)

Recrystallized from DMF as yellow crystals, yield: (74%), m.p. 254–255 °C; IR (KBr): v/cm^−1^ 1712, 1685 (2CO); ^1^H-NMR (DMSO-*d*_*6*_): *δ* = 3.90 (s, 3H, O*CH*_*3*_), 3.98 (s, 3H, O*CH*_*3*_), 6.76 (d, *J* = 9.0 Hz, 2H, Ar-H), 7.54-7.64 (m, 2H, Ar-H), 7.90 (s, 1H, Ar-H), 8.18 (d, *J* = 8.4 Hz, 1H, Ar-H), 8.25 (s, 1H, olefinic *CH*) and 9.00 ppm (d, *J* = 8.4 Hz, 1H, Ar-H); MS (EI): *m/z* (%) 367 (M^+^+1, 24.15), 366 (M^+^, 100); HRMS (EI): *m/z* calcd. for C_19_H_14_N_2_O_4_S (M^+^) 366.0668, found 336.0668.

#### (*Z*)-Methyl-2-[5-(4-nitrobenzylidene)-2-imino-4-oxothi-azolidin-3-yl]benzoate (D [Ar = *P*-NO_2_C_6_ H_4_])

A mixture of methyl-2-(2-thiocyanatoacetamido)benzoate (**4**) (1.25 g, 5 mmol) and 4-nitrobenzylidene malononitrile (1.0 g, 5 mmol) in ethanol (25 mL) containing DABCO (0.11 g, 10 mol %) were stirred at reflux for just dissolving the reaction mixture and the product began to separated from the reaction approximately after 2 min. Then, the reaction mixture was filtered off while it hot, the precipitate is **9** and the filtrate containing the intermediate **D** which allowed to cooled to room temperature. The precipitate which formed was filtered off and washed with water and recrystallized from dioxane as pale yellow crystals: m.p. 282–283°C; IR (KBr): v/cm^−1^ 3267 (NH), 1717, 1705 (2CO); ^1^H-NMR (DMSO-*d*_*6*_): *δ* = 3.74 (s, 3H, CH_3_), 7.55 (d, *J* = 8.0 Hz, 1H, Ar-H), 7.66 (t, *J* = 8.0 Hz, 1H, Ar-H), 7.80-7.83 (m, 2H, 1 Ar-H and olefinic *CH*), 7.88 (d, *J* = 8.4 Hz, 2H, Ar-H), 8.07 (d, *J* = 8.0 Hz, 1H, Ar-H), 8.40 (d, *J* = 8.4 Hz, 2H, Ar-H) and 9.97 ppm (s, 1H, NH); ^13^C-NMR (DMSO-*d*_*6*_ at 80°C ): *δ* = 66.3 (CH_3_), 124.3, 125.8, 127.8, 127.8, 129.5, 130.6, 131.0, 131.1, 133.6, 134.5, 139.9, 147.1, 151.9, 164.5 and 165.3 ppm (Ar-C and CO); MS (EI): *m/z* (%) 384 (M^+^+1, 16.25), 383 (M^+^, 4.95); HRMS (EI): *m/z* calcd. for C_18_H_13_N_3_O_5_S (M^+^) 383.0570, found 383.0569.

#### Ethyl-2-[(4-oxo-3,4,-dihydroquinazolin-2-yl)thio]acetate (10)

A solution of methyl-2-(2-thiocyanatoacetamido)benzoate (**4**) (2.50 g, 10 mmol) in ethanol (40 mL) containing DABCO (0.11 g, 10 mol %), was refluxed for 3 h and allowed to cool. The formed precipitate was filtered off, washed with water and then recrystallised from EtOH as white crystals, yield: (99%) {lit. [45] (72%)}, m.p. 184–185°C {lit. [45], mp 179–180°C (MeOH)}; IR (KBr): v/cm^−1^ 3167 (NH), 1728, 1697 (2CO); ^1^H-NMR (DMSO-*d*_*6*_): *δ* = 1.20 (t, 3H, *J* = 7.2 Hz, *CH*_*3*_CH_2_), 4.08 (s, 2H, CH_2_), 4.14 (q, 2H, *J* = 7.2 Hz, CH_3_*CH*_*2*_), 7.40-7.44 (m, 2H , Ar-H), 7.75 (t, *J* = 8.0 Hz, 1H, Ar-H), 8.03 (d, *J* = 8.0 Hz, 1H, Ar-H) and 12.71 ppm (s, 1H, NH); ^13^C-NMR (DMSO-*d*_*6*_): *δ* = 14.1 ( CH_3_), 32.3 (CH_2_), 61.1 (CH_2_), 119.8, 125.8, 125.9, 126.1, 134.3, 148.1, 154.7, 161.1 and 168.4 ppm (Ar-C and CO); MS (EI): *m/z* (%) 265 (M^+^+1, 14.25), 264 (M^+^, 87.44); HRMS (EI): *m/z* calcd. for C_12_H_12_N_2_O_3_S (M^+^) 264.0563, found 264.0563. Crystal data: C_12_H_12_N_2_O_3_S, M = 264.31, orthorhombic, a = 10.1158(2) Å, b = 7.6750(2) Å, c = 31.3650(6) Å, V = 2435.14(9) Å^3^, α = β = γ = 90.0°, space group: P b c a, Z = 8, D_calc_ = 1.442 Mg cm^−3,^ No. of reflections measured 8543, θ_max_ = 66.55°, R1 = 0.034. Figure 9 illustrates the structure as determined. Full data can be obtained on request from the CCDC [54].

#### General procedure for the reaction of 10 with aromatic aldehydes

A mixture of ethyl-2-[(4-oxo-3,4,-dihydroquinazolin-2-yl)thio]acetate (**10**) (1.32 g, 5 mmol) and the appropriate arylaldehyde (5 mmol) in acetic acid (25 mL) containing anhydrous sodium acetate (0.84 g, 10 mmol) was refluxed for 5 h. The reaction mixture was then cooled to room temperature. The precipitate which formed was filtered off and washed with water and the resulting crude product was purified by recrystallization from the appropriate solvent to afford a mixture from **9** and **11** in ratio 1:1 as illustrated from the ^1^H-NMR spectra. We cannot separate the mixtures by crystallization or by long column chromatography due to the difficult solubility of the mixtures so the reported spectral data are for both products (cf. Additional file 1).

#### (*Z*)-2-(4-Methylbenzylidene)-2*H*-thiazolo[2,3-*b*]quinazoline-3,5-dione (11a) and 9b

Recrystallized from dioxane as pale yellow crystals, yield: (38% **9b** + 38% **11a**), m.p. (for mixture) 218–220°C; IR (KBr): v/cm^−1^ 1766, 1727, 1704, 1678 (4CO); ^1^H-NMR (DMSO-*d*_*6*_): *δ* = 2.37 (s, 6H, 2CH_3_), 7.38-7.41 (m, 4H, Ar-H), 7.54 (t, *J* = 7.6 Hz, 1H, Ar-H), 7.58-7.67 (m, 6H, Ar-H), 7.86 (t, *J* = 7.6 Hz, 1H, Ar-H), 7.93 (t, *J* = 8.0 Hz, 1H, Ar-H), 7.96 (s, 1H, olefinic *CH* for 11a), 8.12 (s, 1H, olefinic *CH* for 9b), 8.17 (t, *J* = 7.6 Hz, 2H, Ar-H) and 8.98 ppm (d, *J* = 8.0 Hz, 1H, Ar-H); MS (EI): *m/z* (%) 321(M^+^+1, 21.55), 320 (M^+^, 100); HRMS (EI): *m/z* calcd. for C_18_H_12_N_2_O_2_S (M^+^) 320.0613, found 320.0614.

#### (*Z*)-2-(4-Methoxybenzylidene)-2*H*-thiazolo[2,3-*b*]-quinazoline-3,5-dione (11b) and 9c

Recrysta- llized from dioxane as yellow crystals, yield: (35% **9c** + 35% **11b**), m.p. (for mixture) 206–208°C; IR (KBr): v/cm^−1^ 1765, 1719, 1704, 1683 (2CO); ^1^H-NMR (DMSO-*d*_*6*_): *δ* = 3.84 (s, 6H, 2O*CH*_*3*_), 7.12-7.15 (m, 4H, Ar-H), 7.53 (t, *J* = 8.0 Hz, 1H, Ar-H), 7.60 (t, *J* = 7.6 Hz, 1H, Ar-H), 7.64-7.67 (m, 3H, Ar-H), 7.71 (d, *J* = 8.4 Hz, 2H, Ar-H), 7.85 (t, *J* = 8.0 Hz, 1H, Ar-H), 7.91 (t, *J* = 8.4 Hz, 1H, Ar-H), 7.95 (s, 1H, olefinic *CH* for 11b), 8.10 (s, 1H, olefinic *CH* for 9c), 8.14 (t, *J* = 7.6 Hz, 2H, Ar-H) and 8.98 ppm (d, *J* = 8.4 Hz, 1H, Ar-H); MS (EI): *m/z* (%) 337 (M^+^+1, 22.15), 336 (M^+^, 100); HRMS (EI): *m/z* calcd. for C_18_H_12_N_2_O_3_S (M^+^) 336.0563, found 336.0562.

#### (*Z*)-2-(4-Chlorobenzylidene)-2*H*-thiazolo[2,3-*b*]quinazoline-3,5-dione (11c) and 9d

Recrystallized from dioxane as pale yellow crystals, yield: (34% **9d** + 34% **11c**), m.p. (for mixture) 250–252°C; IR (KBr): v/cm^−1^ 1765 (br), 1698, 1679 (4CO); ^1^H-NMR (DMSO-*d*_*6*_): *δ* = 7.52-7.58 (m, 2H, Ar-H), 7.61-7.69 (m, 6H, Ar-H), 7.74 (d, *J* = 8.4 Hz, 2H, Ar-H), 7.79 (d, *J* = 8.8 Hz, 2H, Ar-H), 7.88 (t, *J* = 8.0 Hz, 1H, Ar-H), 7.95 (t, *J* = 8.4 Hz, 1H, Ar-H), 8.02 (s, 1H, olefinic *CH* for11c), 8.16-8.20 (m, 3H, 2 Ar-H and olefinic *CH* for 9d) and 8.98 ppm (d, *J* = 8.4 Hz, 1H, Ar-H); MS (EI): *m/z* (%) 342 (M^+^+2, 34.66), 341 (M^+^+1, 1985), 340 (M^+^, 100); HRMS (EI): *m/z* calcd. for C_17_H_9_ClN_2_O_2_S (M^+^) 340.0067, found 340.0067.

#### General procedure for the synthesis of compounds 12 and 13

A solution of ethyl-2-[(4-oxo-3,4,-dihydroquinazolin-2-yl)thio]acetate (**10**) (2.64 g, 10 mmol), *N,N*-dimethylformamide dimethylacetal (DMF-DMA) (1.2 g, 10 mmol) in ethanol (30 mL) were stirred at reflux for 6 h. The separated solid product obtained on standing at room temperature was collected by filtration, washed by EtOH and recrystallized from EtOH the dissolved product was identified as **12** and the undissolved one recrystalized from DMSO and identified as **13**.

#### 5-Oxo-5*H*-thiazolo[2,3-*b*]quinazoline-2-carboxylic acid ethyl ester (12)

Yield: 43%, m.p. 293–294°C; IR (KBr): v/cm^−1^ 1727, 1691 (2CO); ^1^H-NMR (DMSO-*d*_*6*_): *δ* = 1.34 (t, 3H, *J* = 7.2 Hz, *CH*_*3*_CH_2_), 4.37 (q, 2H, *J* = 7.2 Hz, CH_3_*CH*_*2*_), 7.55 (t, *J* = 8.0 Hz, 1H, Ar-H), 7.66 (d, *J* = 8.0 Hz, 1H, Ar-H), 7.92 (t, *J* = 8.0 Hz, 1H, Ar-H), 8.25 (d, *J* = 8.0 Hz, 1H, Ar-H) and 8.44ppm (s, 1H, thiazole *H3*); *m/z* (%) 275 (M^+^+1, 16.46), 274 (M^+^, 100); HRMS (EI): *m/z* calcd. for C_13_H_10_N_2_O_3_S (M^+^) 274.0406, found 274.0406. Crystal data: C_13_H_10_N_2_O_3_S, M = 274.30, monolinic, a = 10.555(4) Å, b = 25.556(7) Å, c = 9.115(3) Å, V = 2455(2) Å^3^, α = γ = 90.00°, β =93.191(7)^o^, space group: C2/c (#15), Z = 8, D_calc_ = 1.484 Mg cm^−3^ , No. of reflections measured 2482, 2θ_max_ = 52.70°, R1 = 0.0666. Figure 10 illustrates the structure as determined. Full data can be obtained on request from the CCDC [55].

#### (*Z*)-2-[(Dimethylamino)methylene]-2*H*-thiazolo[3,2-*a*]-quinazoline-1,5-dione (13)

Yield: 43%, m.p. 277–278°C; IR (KBr): v/cm^−1^ 1706, 1692 (2CO); ^1^H-NMR (DMSO-*d*_*6*_): *δ* = 3.29 (s, 6H, 2CH_3_), 7.61 (t, *J* = 7.6 Hz, 1H, Ar-H), 7.85 (t, *J* = 7.6 Hz, 1H, Ar-H), 8.08 (s, 1H, olefinic *CH*), 8.15 (d, *J* = 7.6 Hz, 1H, Ar-H) and 9.21 ppm (d, *J* = 7.6 Hz, 1H, Ar-H); *m/z* (%) 274 (M^+^+1, 19.88), 273 (M^+^, 100); HRMS (EI): *m/z* calcd. for C_13_H_11_N_3_O_2_S (M^+^) 273.0566, found 273.0567. Crystal data: C_13_H_11_N_3_O_2_S, M = 273.32, monolinic, a = 23.074(2) Å, b = 10.1009(7) Å, c = 13.896(1)Å, V = 676.2(9) Å^3^, α = γ = 90.00°, β =105.058(8)^o^, space group: C2/c (#15), Z = 8, D_calc_ = 1.493Mg cm^−3^ , No. of reflections measured 3557, 2θ_max_ = 54.90°, R1= 0.0381. Figure 11 illustrates the structure as determined. Full data can be obtained on request from the CCDC [56].

#### 2-[(4-Oxo-3,4-dihydroquinazolin-2-yl)thio]acetohydrazide (14)

A solution of ethyl-2-[(4-oxo-3,4,-dihydroquinazolin-2-yl)thio]acetate (**10**) (2.64 g, 10 mmol), hydrazine hydrate 99% (0.75 g, 15 mmol) in absolute ethanol (30 mL) was stirred at reflux for 2 h. The separated solid product from the reaction mixture was collected by filtration, washed by water and recrystallized from EtOH as white crystals, yield: 92%, m.p. above 300°C; IR (KBr): v/cm^−1^ 3432, 3305, 3295, 3237 (2NH and NH_2_), 1685, 1651(2CO); ^1^H-NMR (DMSO-*d*_*6*_): *δ* = 3.93 (s, 2H, CH_2_), 4.34 (br, 2H, NH_2_, D_2_O exchangeable ), 7.43 (t, *J* = 8.0 Hz, 1H, Ar-H), 7.53 (d, *J* = 8.0 Hz, 1H, Ar-H), 7.77 (t, *J* = 8.0 Hz, 1H, Ar-H), 8.03 (d, *J* = 8.0 Hz, 1H, Ar-H), 9.37 (br, 1H, NH) and 12.71 ppm (s, 1H, NH); ^13^C-NMR (DMSO-*d*_*6*_): *δ* = 32.1 (CH_2_), 119.9, 125.7, 126.0, 134.6, 148.2, 155.2, 161.2, 166.5 and 171.1 ppm (Ar-C and CO); MS (EI): *m/z* (%) 251 (M^+^+1, 4.22), 250 (M^+^, 13.89); HRMS (EI): *m/z* calcd. for C_10_H_10_N_4_O_2_S (M^+^) 250.0518, found 250.0519.

#### (*E*)-*N'-*(4-Chlorobenzylidene)-2-[(4-oxo-3,4-dihydro-quinazolin-2-yl)thio]acetohydrazide (15)

A solution of the acetohydrazide **14** (1.25 g, 5 mmol), 4-chlorobenzaldehyde (0.70 g, 5 mmol) in ethanol (25 mL) containing DABCO (0.11 g, 10 mol %) was stirred at reflux for 5 h. The separated solid product obtained on standing at room temperature was collected by filtration, washed by EtOH and recrystallized from dioxane/DMF (1:1) mixture as white crystals, yield: 77%, m.p. 234–235 °C; IR (KBr): v/cm^−1^ 3438, 3173 (2NH), 1673 (br 2CO); ^1^H-NMR (DMSO-*d*_*6*_): *δ* = 3.57 (s, 2H, CH_2_), 7.40-7.52 (m, 4H, Ar-H), 7.72-7.76 (m, 3H, Ar-H), 8.02-8.05 (m, 2H, 1Ar-H and amidine *CH*), 11.74 (br, 1H, NH) and 12.70 ppm (s, 1H, NH); ^13^C-NMR (DMSO-*d*_*6*_): *δ* = 31.8 (CH_2_), 119.9, 125.9, 126.0, 128.5, 128.9, 133.0, 134.6, 142.1, 145.4, 148.2, 155.1, 161.1, 163.8 and 169.0 ppm (Ar-C and CO); MS (EI): *m/z* (%) 373 (M^+^+1, 6.18), 372 (M^+^, 24.79); HRMS (EI): *m/z* calcd. for C_17_H_13_ClN_4_O_2_S (M^+^) 372.0442, found 372.0442.

#### *N-*(4-Chlorophenyl)-2-[(4-oxo-3,4-dihydroquinazolin-2-yl)-thio]acetamide (16)

A solution of ethyl-2-[(4-oxo-3,4,-dihydroquinazolin-2-yl)thio]acetate (**10**) (1.32 g, 5 mmol), 4-chloro-aniline (0.70 g, 5 mmol) in acetic acid (25 mL) was stirred at reflux for 5 h. The separated solid product obtained on standing at room temperature was collected by filtration, washed by EtOH and recrystallized from dioxane as white crystals, yield: 84%, m.p. 276–278°C; IR (KBr): v/cm^−1^ 3406, 3185 (2NH), 1687 (br 2CO); ^1^H-NMR (DMSO-*d*_*6*_): *δ* = 3.38 (s, 2H, CH_2_), 7.25 (t, *J* = 7.6 Hz, 1H, Ar-H), 7.39-7.42 (m, 3H, Ar-H), 7.67 (t, *J* = 7.6 Hz, 1H, Ar-H), 7.78 (d, *J* = 8.0 Hz, 2H, Ar-H), 7.98 (d, *J* = 7.6 Hz, 1H, Ar-H), 8.83 (s, 1H, NH) and 10.87 ppm (s, 1H, NH); ^13^C-NMR (DMSO-*d*_*6*_): *δ* = 24.0 (CH_2_), 118.4, 120.8, 123.2, 125.3, 125.9, 128.6, 134.4, 138.0, 147.2, 149.7, 161.6, 168.4 and 172.0 ppm (Ar-C and CO); MS (EI): *m/z* (%) 346 (M^+^+1, 3.74), 345 (M^+^, 11.25); HRMS (EI): *m/z* calcd. for C_16_H_12_^35^ClN_3_O_2_S (M^+^) 345.0333, found 345.0331.

#### *2-*[(4-Amino-5-mercapto-4*H*-1,2,4-triazol-3-yl)methylthio]-3*H*-quinazolin-4-one (18)

Carbon disulphide (20 mmol) was added drop wise to an ice cold solution of potassium hydroxide (10 mmol) in absolute alcohol (30 ml) containing acetohydrazide **14** (1.25 g, 5 mmol). The reaction mixture was stirred continuously for 24 h at room temperature. The precipitated potassium thiocarbamate **17** was filtered off, washed with chilled diethyl ether then dried and directly used for the next step without further purification. The above potassium thiocarbamate was mixed with water (8 mL) and hydrazine hydrate (15 mmol) and refluxed for 4 h. The reaction mixture turned green with evolution of hydrogen sulphide and finally it became homogeneous. The reaction mixture was cooled to room temperature and poured onto ice cold water. On acidification with acetic acid, the required triazole **18** was precipitated then filtered off and washed with cold water and dried. It was purified by recrystallization from EtOH/DMF (1:2) mixture to get white, crystalline solid. yield: 66%, m.p. 204–205°C; IR (KBr): v/cm^−1^ 3327, 3301, 3263, 3202 (2NH and NH_2_), 1665 (CO); ^1^H-NMR (DMSO-*d*_*6*_): *δ* = 4.40 (s, 2H, CH_2_), 5.43(s, 2H, NH_2_ D_2_O exchangeable ), 7.14 (t, *J* = 8.0 Hz, 1H, Ar-H), 7.32 (d, *J* = 8.0 Hz, 1H, Ar-H), 7.60 (t, *J* = 8.0 Hz, 1H, Ar-H), 7.93 (d, *J* = 8.0 Hz, 1H, Ar-H), 12.85 (s, 1H, NH) and 13.87 ppm (s, 1H, NH); ^13^C-NMR (DMSO-*d*_*6*_): *δ* = 32.3 (CH_2_), 116.5, 121.1, 121.6, 124.4, 126.1, 134.0, 148.6, 152.6, 161.0 and 166.5 ppm (Ar-C and CO); MS (EI): *m/z* (%) 307 (M^+^+1, 21.62), 306 (M^+^, 100); HRMS (EI): *m/z* calcd. for C_11_H_10_N_6_OS_2_ (M^+^) 306.0352, found 306.0354.

#### *N-*(2-Hydroxyethyl)-2-[(4-oxo-3,4-dihydroquinazolin-2-yl)-thio]acetamide (19)

Mixture of ethyl-2-[(4-oxo-3,4,-dihydroquinazolin-2-yl)thio]acetate (**10**) (2.64 g, 10 mmol), ethanolamine (1.22 g, 20 mmol) in ethanol (25 mL) was stirred at reflux for 4 h. The separated solid product obtained on standing at room temperature was collected by filtration, washed by water and recrystallized from EtOH as colorless crystals, yield: 71%, m.p. 228–229°C; IR (KBr): v/cm^−1^ 3385, 3267, 3202 (2NH and OH), 1689, 1629 (2CO); ^1^H-NMR (DMSO-*d*_*6*_): *δ* = 3.35 (s, 2H, CH_2_), 3.40 (q, 2H, *J* = 5.6 Hz, *CH*_*2*_OH), 3.56 (t, 2H, *J* = 5.6 Hz, NH*CH*_*2*_), 4.94 (br, 1H,OH, D_2_O exchangeable), 6.37 (s, 1H,NH), ), 7.10 (t, *J* = 8.0 Hz, 1H, Ar-H), 7.24 (d, *J* = 8.0 Hz, 1H, Ar-H), 7.56 (t, *J* = 8.0 Hz, 1H, Ar-H), 7.88 (d, *J* = 8.0 Hz, 1H, Ar-H) and 9.86 ppm (s, 1H, NH); MS (EI): *m/z* (%) 280 (M^+^+1, 3.88), 279 (M^+^, 15.09); HRMS (EI): *m/z* calcd. for C_12_H_13_N_3_O_3_S (M^+^) 279.0672, found 279.0671.

## Conclusions

In conclusion a simple and efficient one-pot synthesis of a novel class of 2-arylidene-2*H*-thiazolo[3,2-*a*]-quinazoline-1,5-diones **9a-i** was established through DABCO catalyzed Michael type addition reaction. In addition many fused quinazoline and quinazoline derivatives were synthesized which appeared as valuable precursors in synthetic and medicinal chemistry. Moreover the X-ray single crystal technique was successfully employed in this study for structure elucidation, Z/E potential isomerism configuration determination and to determine the regioselectivity of the reactions.

## Competing interests

The authors declare that they have no competing interests.

## Authors’ contributions

The current study is an outcome of the constructive discussion and work between HB and HMI, who carried out the synthesis, purification and characterization of the compounds by the different analysis tools such as the HRMS, ^1^H NMR, ^13^C NMR spectral analyses and the X-ray single crystal analysis. Both HB and HMI prepared, read and approved the final manuscript.

## Supplementary Material

Additional file 1 ^1^H-NMR and FT-IR spectra of compounds 11a plus 9b and 11b plus 9c. Click here for file
