# Supplementary material for: Organocatalysis in heterocyclic synthesis: DABCO as a mild and efficient catalytic system for the synthesis of a novel class of quinazoline, thiazolo [3,2-a]quinazoline and thiazolo[2,3-b] quinazoline derivatives
Source: Chem Cent J. 2013 May 7;7:82. doi: 10.1186/1752-153X-7-82 (PMC3681655; doi:10.1186/1752-153X-7-82)
Supplement: Additional file 1 — 1H-NMR and FT-IR spectra of compounds 11a plus 9b and 11b plus 9c. [file 1752-153X-7-82-S1.pdf]

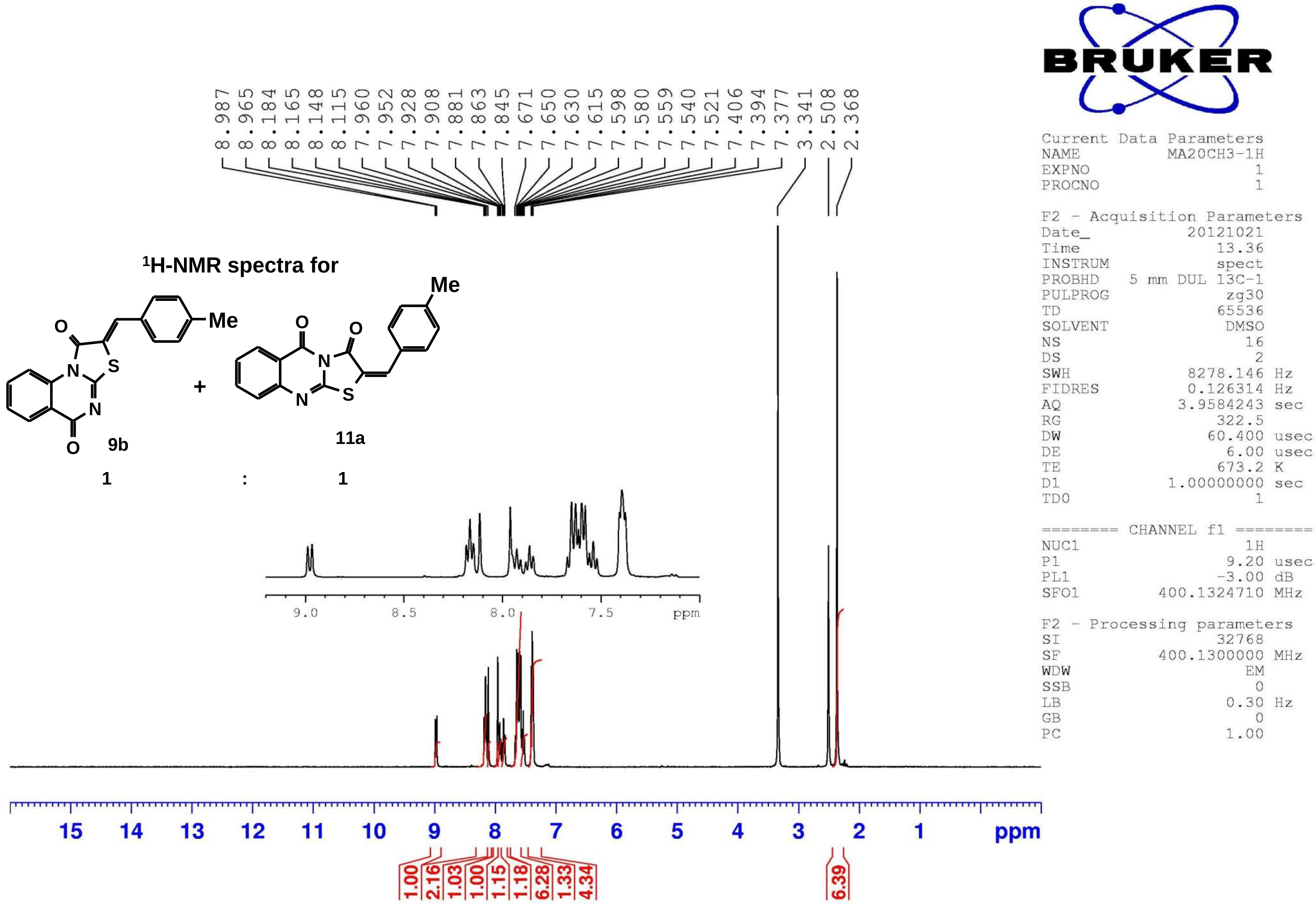

1H-NMR spectra for
9b
11a
1 : 1
8.987 8.965 8.184 8.165 8.148 8.115 7.960 7.952 7.928 7.908 7.881 7.863 7.845 7.671 7.650 7.630 7.615 7.598 7.580 7.559 7.540 7.521 7.406 7.394 7.377 3.341 2.508 2.368
BRUKER
Current Data Parameters
NAME MA20CH3-1H
EXPNO 1
PROCNO 1
F2 - Acquisition Parameters
Date_ 20121021
Time 13.36
INSTRUM spect
PROBHD 5 mm DUL 13C-1
PULPROG zg30
TD 65536
SOLVENT DMSO
NS 16
DS 2
SWH 8278.146 Hz
FIDRES 0.126314 Hz
AQ 3.9584243 sec
RG 322.5
DW 60.400 usec
DE 6.00 usec
TE 673.2 K
D1 1.00000000 sec
TD0 1
======== CHANNEL f1 ========
NUC1 1H
P1 9.20 usec
PL1 -3.00 dB
SFO1 400.1324710 MHz
F2 - Processing parameters
SI 32768
SF 400.1300000 MHz
WDW EM
SSB 0
LB 0.30 Hz
GB 0
PC 1.00
1.00 2.16 1.03 1.00 1.15 1.18 6.28 1.33 4.34 6.39
ppm

# General Facility Projects - GS01/05
# Kuwait University

## FT-IR Spectral Data

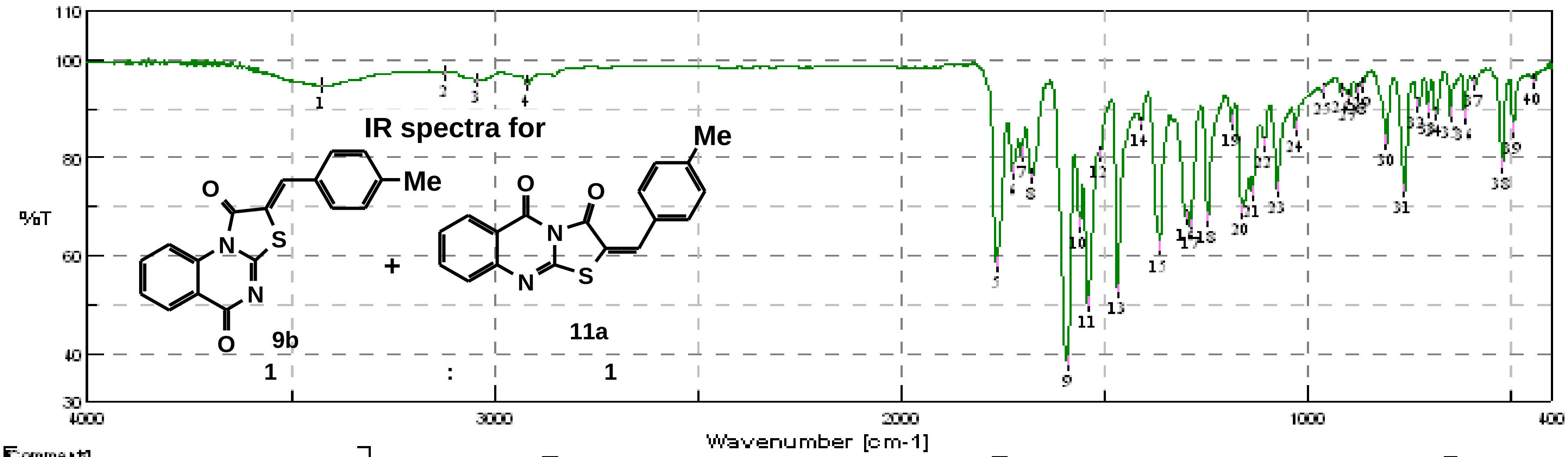


[Comments]

| | |
|---|---|
| Sample name | MA 20 CH3 CRUDE |
| Comment | PREPARED ON 23.1.13 |
| User | DR HEIDER |
| Division | GFS GS 01/05 |
| Company | KU |

[Measurement Information]

| | |
|---|---|
| Model Name | FT/IR-6300typeA |
| Serial Number | A009861024 |
| Light Source | Standard |
| Detector | TGS |
| Accumulation | Auto (31) |
| Resolution | 4 cm-1 |
| Zero Filling | On |
| Apodization | Cosine |
| Gain | Auto (2) |
| Aperture | Auto (7.1 mm) |
| Scanning Speed | Auto (2 mm/sec) |
| Filter | Auto (10000 Hz) |

Result of Peak Picking

| No. | Position | Intensity | No. | Position | Intensity | No. | Position | Intensity |
|---|---|---|---|---|---|---|---|---|
| 1 | 3423.03 | 94.6758 | 2 | 3123.15 | 97.1652 | 3 | 3045.05 | 95.6675 |
| 4 | 2918.73 | 95.0315 | 5 | 1765.51 | 58.0578 | 6 | 1726.94 | 76.9607 |
| 7 | 1703.8 | 80.4294 | 8 | 1677.77 | 76.1487 | 9 | 1591.95 | 37.7664 |
| 10 | 1561.09 | 66.0902 | 11 | 1540.85 | 49.8121 | 12 | 1510.95 | 80.4788 |
| 13 | 1468.53 | 52.5245 | 14 | 1410.67 | 86.8253 | 15 | 1365.35 | 61.2118 |
| 16 | 1299.79 | 67.7278 | 17 | 1290.14 | 65.828 | 18 | 1247.72 | 67.1729 |
| 19 | 1187.94 | 86.9996 | 20 | 1162.87 | 68.855 | 21 | 1136.83 | 72.6101 |
| 22 | 1106.94 | 82.4239 | 23 | 1076.08 | 73.2279 | 24 | 1030.77 | 85.8592 |
| 25 | 964.233 | 93.5503 | 26 | 917.95 | 93.5793 | 27 | 899.63 | 92.2835 |
| 28 | 880.345 | 93.3082 | 29 | 866.846 | 94.6776 | 30 | 808.028 | 82.8659 |
| 31 | 762.709 | 73.0555 | 32 | 729.925 | 90.645 | 33 | 704.855 | 89.4158 |
| 34 | 685.57 | 88.8773 | 35 | 650.858 | 88.473 | 36 | 616.145 | 88.2559 |
| 37 | 590.111 | 95.0382 | 38 | 523.579 | 78.0671 | 39 | 495.616 | 85.3191 |
| 40 | 445.476 | 95.4494 | | | | | | |

## $^{1}$H-NMR spectra for

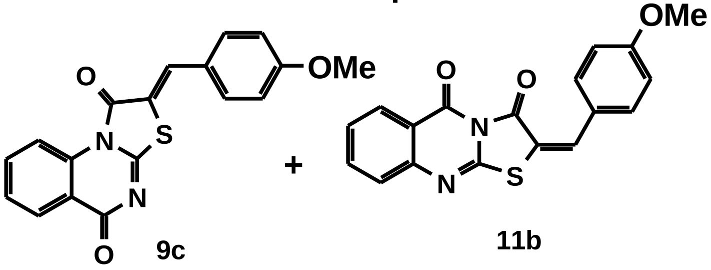


9c + 11b

1 : 1

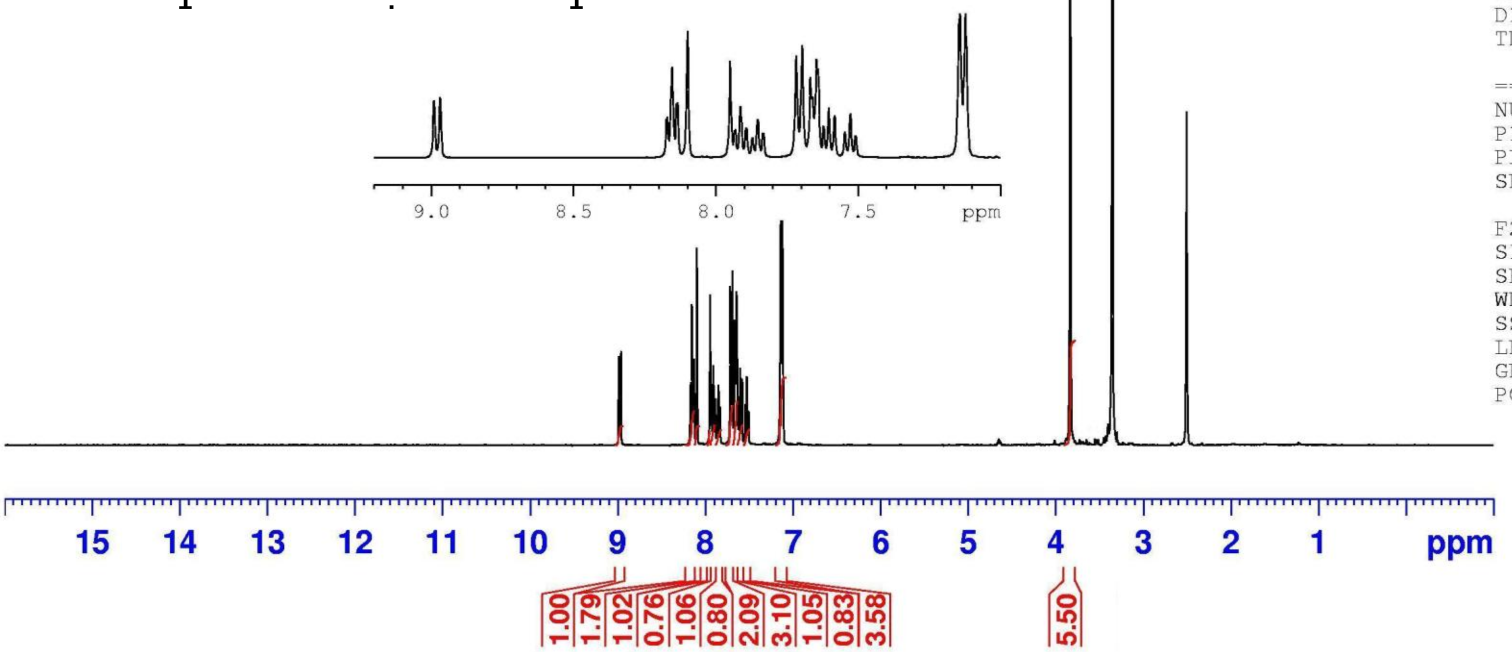


BRUKER

Current Data Parameters
NAME MA17-1H
EXPNO 1
PROCNO 1

F2 - Acquisition Parameters
Date_ 20121017
Time 13.25
INSTRUM spect
PROBHD 5 mm DUL 13C-1
PULPROG zg30
TD 65536
SOLVENT DMSO
NS 16
DS 2
SWH 8278.146 Hz
FIDRES 0.126314 Hz
AQ 3.9584243 sec
RG 287.4
DW 60.400 usec
DE 6.00 usec
TE 673.2 K
D1 1.00000000 sec
TD0 1

======== CHANNEL f1 ========
NUC1 1H
P1 9.20 usec
PL1 -3.00 dB
SFO1 400.1324710 MHz

F2 - Processing parameters
SI 32768
SF 400.1300000 MHz
WDW EM
SSB 0
LB 0.30 Hz
GB 0
PC 1.00

# General Facility Projects - GS01/05
# Kuwait University

## FT-IR Spectral Data

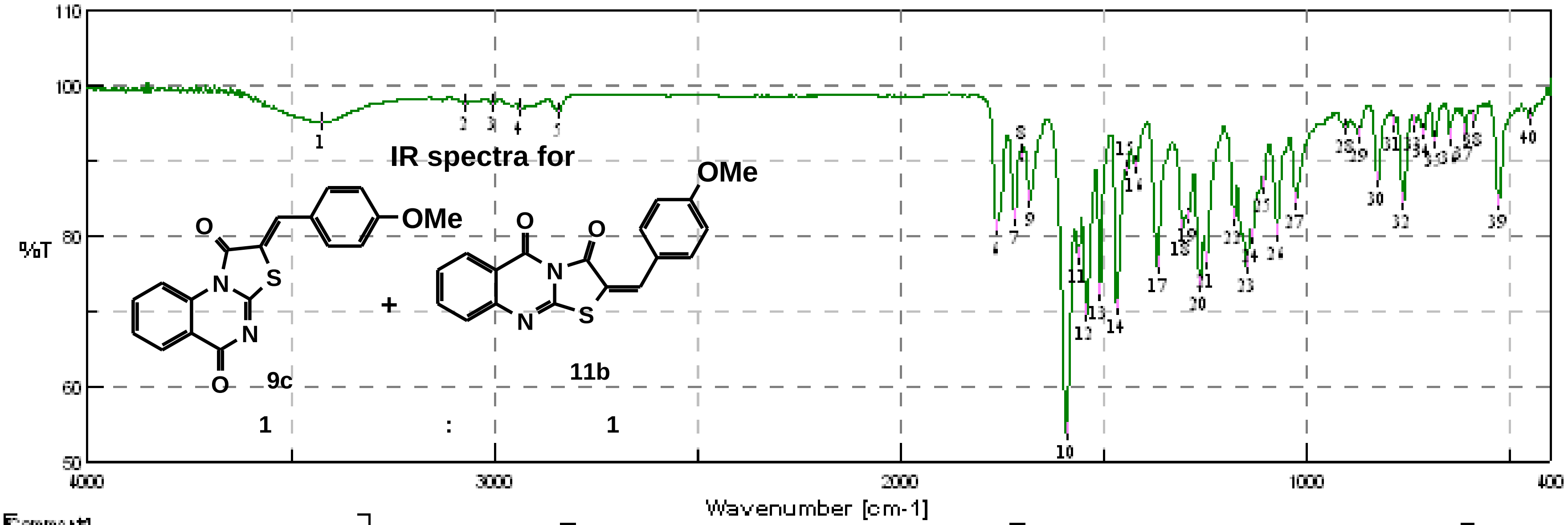


[Comment]

| | |
|---|---|
| Sample name | MA16 |
| Comment | PREPARED ON 22.1.13 |
| User | DR. HEIDER |
| Division | GFS GS 01/05 |
| Company | KU |

[Measurement Information]

| | |
|---|---|
| Model Name | FT/IR-6300typeA |
| Serial Number | A009661024 |
| Light Source | Standard |
| Detector | TGS |
| Accumulation | Auto (25) |
| Resolution | 4 cm-1 |
| Zero Filling | On |
| Apodization | Cosine |
| Gain | Auto (2) |
| Aperture | Auto (7.1 mm) |
| Scanning Speed | Auto (2 mm/sec) |
| Filter | Auto (10000 Hz) |

Result of Peak Picking

| No. | Position | Intensity | No. | Position | Intensity | No. | Position | Intensity |
|---|---|---|---|---|---|---|---|---|
| 1 | 3423.99 | 95.0835 | 2 | 3072.05 | 97.5905 | 3 | 3005.52 | 97.6169 |
| 4 | 2935.13 | 97.0501 | 5 | 2841.6 | 96.5336 | 6 | 1764.55 | 80.8476 |
| 7 | 1719.23 | 82.4664 | 8 | 1703.8 | 90.9746 | 9 | 1682.59 | 84.8852 |
| 10 | 1592.91 | 54.0035 | 11 | 1562.06 | 77.2181 | 12 | 1542.77 | 69.6536 |
| 13 | 1509.99 | 72.3949 | 14 | 1467.56 | 70.5181 | 15 | 1445.39 | 88.9725 |
| 16 | 1422.24 | 89.4993 | 17 | 1368.25 | 76.0267 | 18 | 1307.5 | 80.9812 |
| 19 | 1294.97 | 82.5827 | 20 | 1264.11 | 73.7144 | 21 | 1248.68 | 76.7883 |
| 22 | 1180.22 | 82.366 | 23 | 1149.37 | 76.0551 | 24 | 1135.87 | 79.9129 |
| 25 | 1106.94 | 86.7159 | 26 | 1074.16 | 80.2733 | 27 | 1028.84 | 84.5689 |
| 28 | 904.451 | 94.4153 | 29 | 873.596 | 93.4256 | 30 | 825.384 | 87.4234 |
| 31 | 786.815 | 94.978 | 32 | 763.673 | 84.4788 | 33 | 734.746 | 94.9361 |
| 34 | 714.497 | 93.7989 | 35 | 687.498 | 92.723 | 36 | 649.893 | 93.0329 |
| 37 | 613.252 | 93.6158 | 38 | 589.147 | 95.4332 | 39 | 529.364 | 84.3831 |
| 40 | 454.154 | 95.7093 | | | | | | |
